# Jin-Wu-Jian-Gu Formulation Attenuates Rheumatoid Arthritis by Inhibiting the IL33-ST2 Signaling Pathway

**DOI:** 10.1155/2022/6821388

**Published:** 2022-01-20

**Authors:** Daomin Lu, Ying Huang, Wukai Ma, Changming Chen, Lei Hou

**Affiliations:** ^1^Department of Rheumatology and Immunology, The Second Affiliated Hospital of Guizhou University of Traditional Chinese Medicine, Fei Shan Street, Guiyang 550001, China; ^2^Guizhou Medical University, Beijing Road, Guiyang, Guizhou Province 550004, China

## Abstract

The present research attempted to investigate the molecular mechanism of Jin-Wu-Jian-Gu Formulation (JWJGF) in inhibiting rheumatoid arthritis (RA) in a pharmacological approach for analysis and experimental validation. First, the potential targets and pathways of JWJGF for RA were predicted by network pharmacology. Second, the effect of JWJGF on RA was observed by hematoxylin-eosin (HE) staining and enzyme-linked immunosorbent assay (ELISA). Further, we observed the effects of JWJGF on the IL33-ST2 signaling pathway by Western blot and quantitative real-time PCR (qPCR) experiments, and finally, we studied the effects of Liquiritigenin on rheumatoid arthritis synovial fibroblast (RASF) cells and the IL33-ST2 signaling pathway. Network pharmacology results showed that the key component of JWJGF was Liquiritigenin and the core target of JWJGF was IL-33. The results of HE and ELISA showed that JWJGF could alleviate RA. Western blot and qPCR findings indicated that JWJGF could inhibit the IL33-ST2 signaling pathway. Furthermore, JWJGF could inhibit the proliferation of RASF cells and the IL33-ST2 signaling pathway. In conclusion, this study revealed that JWJGF attenuated RA by inhibiting the IL33-ST2 signaling pathway.

## 1. Introduction

RA represents a frequently encountered chronic systemic disorder in the immune system. It is characterized by infiltration of a large number of inflammatory cells, proliferation of synovial tissue, increase of inflammatory factors, and serious damage of articular cartilage and bone tissue. Abnormal proliferation and inhibition of apoptosis of RASF develop in RA patients, which promotes the development of RA [1–4].

Interleukin- (IL-) 33 is a cytokine of the IL-1 family originating from epithelial cells. The receptor for IL-33, growth stimulation expressed gene 2 (ST2), is expressed in a wide range of cells including immunocytes. ST2 has two main forms: soluble growth stimulation expressed gene 2 (sST2) and transmembrane (ST2). A study has reported that the organism can release intracellular IL-33 under stress and infection, which plays an important role in an allergic reaction, inflammation, autoimmune disease, and host defense [5–7].

JWJGF is a long-term clinical study of the vaccine prescription of this research group. It produces a satisfactory curative effect on RA. JWJGF is composed of *Herba Cibotium barometz* (JM), *Herba homalomena* (QN), *Herba Periploca forrestii Schltr* (HG), *Herba Zaocys dhumnades* (WS), *Herba Sabia parviflora Wall* (XH), and *Herba pseudoginseng* (SQ). Liquiritigenin (LQ) is a common component of SQ and QN. As a dihydroflavonoid compound, LQ exerts certain effects on the cardiovascular system and nervous system. In addition, it functions in antivirus, antioxidant, antitumor, and other pharmacological effects [8–11].

Initially, the possible targets and pathways of JWJGF for RA were predicted using network pharmacology and it was found that the key component of the JWJGF was LQ and the core target of the JWJGF was IL-33. Subsequently, the effect of JWJGF on RA was verified by constructing an RA model. Further, the role and molecular mechanism of JWJGF were investigated through Western blot and qPCR experiments. Finally, we examined the effects of LQ on RASF cells and the IL33-ST2 signaling pathway.

## 2. Methods

### 2.1. Experimental Herbal Formulation

JWJGF is a long-term clinical study of the vaccine prescription of this research group, which has been proved to exert a satisfactory curative effect on RA. Herbs were immersed in 1 000 mL drinking water for 1 h before decoction for 30 min. All of the medicine was decocted with 500 mL water for 30 min. Both were mixed and concentrated to 1 g/mL of the raw medicine by evaporation.

### 2.2. Experimental Animals

Male Sprague Dawley (SD) rats weighing approximately 150–180 g were prepared. Animal experiments were approved and performed as per the guidelines of Committee on the Ethics of Animal Experiments of the Second Affiliated Hospital of Guizhou University of Traditional Chinese Medicine, China. The laboratory animal license is SCXK (Xiang) 2019–0004. The laboratory animals were fed under a condition of 12 h light-dark alternatives with food and water available at will.

The rats were randomly classified into 7 groups as follows: control group, RA model group, RA + prednisone group, RA + GTW group, RA + L-JWJGF group, RA + M-JWJGF group, and RA + H-JWJGF group (*n* = 15/group). Complete Freund's adjuvant at 0.1 mL was commenced by intradermal administration into the left posterior metatarsal footpad to induce arthritis. On day 12 following model establishment, the rats in the control group were given intradermal injections while the remaining groups were given 0.5% sodium carboxymethylcellulose intragastrically. The animals were treated with prednisone (50 mg/kg) and glycosides of *Tripterygium wilfordii* (GTW, 100 mg/kg) by gavage. The low-dose JWJGF group was administered at a dose of 5 g/kg, the medium-dose JWJGF group of 10 g/kg, and the high-dose JWJGF group of 20 g/kg, and all were given by gavage. The treatment of the three groups lasted for four weeks.

### 2.3. Cell Cultures and Experimental Treatments

RASF cells were bought from American Type Culture Collection (ATCC) (Manassas, USA), grown in DMEM by addition of 10% fetal bovine serum and followed by administration of Liquiritigenin (50, 100, and 150 *μ*M), prednisone (200 ng/mL), and glycosides of *Tripterygium wilfordii* (30 *μ*g/mL) for 24 h.

### 2.4. Network Pharmacology Analysis

Targets of active components contained in JWJGF were retrieved from website (http://www.swisstargetprediction.ch/). The human RA genes were screened out from GeneCards (https://www genecards.org/), a disease database. A PPI network diagram was created and exported from String (https://string-db.org/), and a drug-component-target network was constructed via Cytoscape 3.6.0. The GO analysis and KEGG pathway analysis were performed on target sites using ClueGO, a plugin of the Cytoscape software, and the enrichment analysis results were visualized ultimately.

### 2.5. Western Blot Analysis

Cells were collected and added to radio-immunoprecipitation assay (RIPA) lysate including PMSF and proteinase inhibitor cocktail. Following treatment at 4°C and 13 000 rpm centrifugation for 20 min, the supernatant was transferred into a precooled 1.5 mL centrifuge tube for concentration determination. Total protein was added at a quantity of 30 *μ*g/well, boiled at 100°C for 10 min, separated by 10% polyacrylamide gel electrophoresis (SPS-PAGE), and followed by transference to a phenylmethanesulfonyl fluoride (PDVF) membrane. Following being sealed with 5% skimmed milk for 1 h at room temperature, the membrane was rinsed 3 times using TBST, 5 min each time. BSA primary antidilution solution at 3% was diluted with 1 : 1 000∼2 000, incubated at 4°C overnight, and incubated by supplemented secondary antidilution solution (1 : 1 000) for 1 h on the next day.

### 2.6. qPCR Assay

Total RNA extraction was subsequently performed using Trizol reagent (Takara, Japan) as per the instructions of use. The reverse transcription of CDNA was conducted using PrimeScript TMR *T* kit (Takara, Japan). The response system and procedures for qRT-PCR were conducted as described in the instructions of TB Green Premix Ex Taq II (Takara, Japan), and a real-time system employed CFX96 (Bio-Rad, USA). Relative expression levels of genes were calculated using the 2^−∆∆CT^ algorithm. Primer sequence of each gene was as follows: sST2-F(GGTGTGACCGACAAGGACT), sST2-R(TTGTGAGAGACACTCCTTAC), ST2L-F(AGTTGTGCATTTACGGGAGAG), ST2L-R(GGATACTGCTTTCCACCACAG), IL-33-F(GTGCAGGAAAGGAAGACTCG), IL-33-R(TGGCCTCACCATAAGAAAGG), GAPDH-F(GCAAGTTCAACGGCACAG), and GAPDH-R(GCCAGTAGACTCCACGACATA).

### 2.7. EDU Assay

RASF cells were inoculated into a 24-well plate. According to the instructions of the EdU kit, 2 × EdU reaction solution was prepared and added to a 24-well plate. The cells were incubated in the reaction solution for 2 h in the dark, fixed by 4% paraformaldehyde for 20 min at room temperature, and supplemented with 500 *μ*L 0.3% Triton X-100. After reaction for 10 min at room temperature, the cells were rinsed 3 times using PBS. AZIDE 555-Click reaction solution was prepared, and 200 *μ*L was added to each well and incubated in the dark at room temperature for 30 min. Following the removal of the reaction solution and three cycles of washing with PBS, the nucleus was restained by Hoechst, and immunofluorescence was conducted subsequently.

### 2.8. TUNEL Assay

The slides were fixed with paraformaldehyde at room temperature for 15–30 min and followed by three cycles of washing with PBS. A sealing medium was subsequently supplied for cell culture at room temperature for 10 min. After rinsing with PBS, the cells were added with a membrane-penetrating solution and incubated at room temperature for 30 min. The TUNEL reaction mixture was prepared and then mixed with 50 *μ*L TdT+450 *μ*L fluorescein-labeled dUTP solution. Reaction time was set as 30 min at room temperature. The cells were added with 50 *μ*L TUNEL reaction mixture, and reacted in a dark wet chamber at 37°C for 60 min. The apoptotic cells were counted under a fluorescence microscope with 1 drop of PBS.

### 2.9. HE Staining

Freshly prepared paraformaldehyde (4%) was employed to fix the samples and sections were embedded in paraffin as per routine histological procedures. After that, the sections were made into 4.5 *μ*m thickness and ready for hematoxylin and eosin (HE) staining. Following the scanning of the stained slides using Pannoramic Scan 250 Flash, photographs were captured using Pannoramic Viewer.

### 2.10. ELISA Assay

Quantification of inflammatory cytokines TNF-*α*, TNF-*γ*, IL-1, IL-5, IL-6, IL-13 IL-17, and IL-1*β* in rat serum was detected by ELISA (Kehua Bio-Engineering, Shanghai, China), and all procedures were performed as the description of the manufacturer's instructions.

### 2.11. Statistical Analysis

SPSS 23.0 was used to analyze the data. The mean of both groups of independent samples was compared using *t*-tests, and the mean value of multiple groups of samples was analyzed by one-way ANOVA, *P* < 0.05. The experimental results were expressed as mean value ± standard deviation and each experiment was repeated three times independently.

## 3. Results

### 3.1. Common Targets and Network Visualization of JWJGF and RA

**Figure 1:**
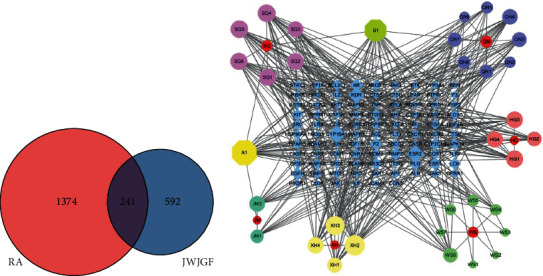
Analysis of key components and core targets of JWJGF therapy for RA based on network pharmacology. (a) Venn diagram of overlapped gene between JWJGF and RA targets. (b) JWJGF-compound-target-RA network diagram. Blue diamonds represented candidate targets of JWJGF against RA, and circular and hexagon shapes represented active ingredients of JWJGF.

To elucidate the potential action mechanism of JWJGF in RA, we first predicted 592 targets of JWJGF active components in the SwissTargetPrediction database, screened 1374 targets of RA-related genes in the GeneCards database, and obtained 241 overlapped genes as the Venn diagram presented (Figure 1(a)). Finally, Cytoscape 3.6 was used to analyze JWJGF, its components, and targets and a drug-active component-target network diagram was plotted. We found that Liquiritigenin had the most connections, and CYP19A1, ESR2, F2, and IL-33 were the potential target proteins (Figure 1(b)). IL-33 was an epithelial cell-derived cytokine found in recent years which could bind with the ST2 receptor to regulate inflammatory factor secretion. There were two secretions of IL-33: active secretion by immune cells and passive excretion by damaged cells. ST2 was a high-affinity receptor for IL-33, and it presented in many immune cells and nonconstructive blood cells. It was found that IL-33 could be released after stress and infection, which played an important role in an allergic reaction, inflammation, autoimmune disease, and host defense.

### 3.2. Effect of JWJGF on the Fibroblast-like Synoviocyte Proliferation and Inflammatory Factors of RA Rats

**Figure 2:**
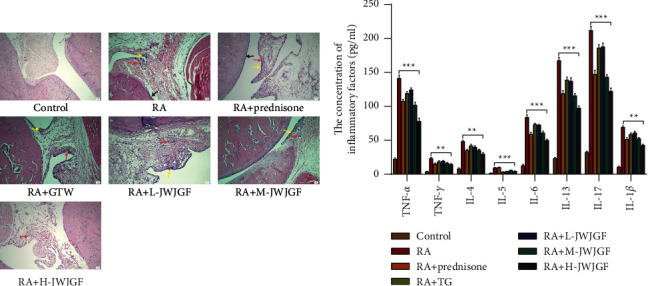
FLS proliferation and inflammatory factors of the RA rat model were suppressed after JWJGF administration. (a) Histological examinations were performed in the hind paw joints (yellow arrows indicated synovial hyperplasia, black arrows indicated chondrocyte injury, and red arrows represented inflammatory cell infiltration. The scale bar was 50 *μ*m). (b) Comparison of various inflammatory factors (TNF-*α*, TNF-*γ*, IL-1, IL-5, IL-6, IL-13, IL-17, and IL-1*β*) of each group using ELISA. Levels of the previously described inflammatory factors were determined after 4 d treatment. All data obtained from experiments were expressed as mean ± SD (*n* > 3). ^*∗*^*p* < 0.05, ^*∗∗*^*p* < 0.01, ^*∗∗∗*^*p* < 0.001, and ^*∗∗∗*^*p* < 0.0001 based on either pairwise comparison using Student's *t*-tests or one-way ANOVA using Tukey's tests for multiple groups.

The main pathological changes of RA are synovial tissue hyperplasia and infiltration of inflammatory cells. The joint synovial tissue cells from the control group were arranged regularly in the absence of both synovial cell hyperplasia and inflammatory cell infiltration. Conversely, hyperemia and edema were recorded in the synovial tissues of RA group. Meanwhile, the apparent proliferation of synovial cells, inflammatory infiltration, and chondrocyte injury was revealed. The hyperemia and edema of synovial tissues in the low- and medium-dose groups of JWJGF decreased slightly, and a small number of synovial cells proliferated inflammatory cells infiltrated. In RA + prednisone group and RA + GTW group, slightly hyperemia, edema, synovial cell proliferation, and a few inflammatory cells infiltration were revealed in the synovial tissues. There was no hyperemia, edema, nor synovial cell proliferation but only a little inflammatory cell infiltration was visualized in the synovial tissues of JWJGF group (Figure 2(a)). There are many inflammatory factors that participated in the pathogenesis of RA. TNF-*α* and IL-1 are recognized as the most important factors, and both of them usually exist at the same time. TNF-*α* and IL-1 are mainly secreted by monocytes and macrophages. TNF-*α* is an important physiological inflammatory mediator. TNF-*α* is secreted at high levels in the active or progressive stage of RA disease, which can cause local joint destruction and corresponding clinical symptoms. At present, it is suggested that the mechanism of TNF-*α* and IL-1*β* inducing joint inflammation is to promote the activation and induction of synovial cells and chondrocytes. Meanwhile, the synthesis of a series of inflammatory mediators, IL-8 and IL-6, produces a powerful proinflammatory effect, leading to synovial inflammation, cartilage destruction, and bone erosion, thereby stimulating the proliferation of synovial fibroblasts. The results of ELISA showed that the expressions of TNF-*α*, TNF-*γ*, IL-1, IL-5, IL-6, IL-13, IL-17, and IL-1*β* in the serum of rats were markedly higher in the model group than those of the control group (*P* < 0.01). Conversely, the serum levels of TNF-*α*, TNF-*γ*, IL-1, IL-5, IL-6, IL-13, IL-17, and IL-1*β* in JWJGF, prednisone, and GTW groups were all decreased to a different extent compared with the control group, and the expression level of Jin-Wu-Jian-Gu decoction in high-dose group was substantially lower than RA group (*P* < 0.01). However, no significant difference was revealed between the prednisone and GTW groups (*P* > 0.05) (Figure 2(b)). The results showed that JWJGF could fight against inflammation by downregulating the inflammatory factors (TNF-*α*, TNF-*γ*, IL-1, IL-5, IL-6, IL-13, IL-17, and IL-1*β*), inhibiting the infiltration of inflammatory cells and the proliferation of synovial tissues, and minimizing destruction of cartilage.

### 3.3. Effect of JWJGF on the IL33-ST2 Signaling Pathway in RA Rats

**Figure 3:**
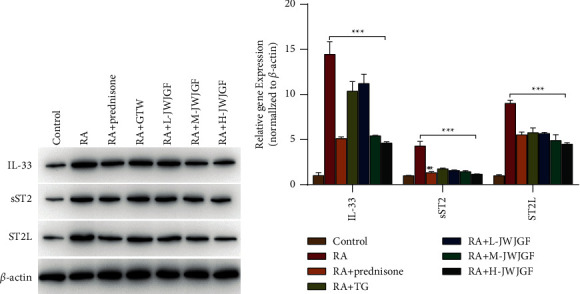
The effects of JWJGF on the IL33-ST2 signaling pathway in RA rats were verified using Western blot and qPCR. (a) Detection of protein expression levels of IL-33, sST2, and ST2L in each group using Western blot. (b) qPCR detection of the relative gene expressions of IL-33, sST2, and ST2L in each group. The data obtained were expressed as mean ± SD (*n* ≥ 3). ^*∗*^*p* < 0.05, ^*∗∗*^*p* < 0.01, ^*∗∗∗*^*p* < 0.001 and ^*∗∗∗∗*^*p* < 0.001 based on either Student's *t*-tests for pairwise comparison or one-way ANOVA using Tukey's tests for multiple group comparison.

The IL33-ST2 signaling pathway exerts a pivotal effect in many diseases. The change of ST2 in the course of disease development has become a hot topic in recent years. SST2 may function as a marker of disease activity in the course of chronic kidney diseases (CKD), and its mechanism in the development of CKD may be the focus of future research. Through network pharmacology, we found that IL-33 could serve as a potential target of JWJGF. To investigate the effect of JWJGF on the IL33-ST2 signaling pathway, we observed the effect of JWJGF on the IL33-ST2 signaling pathway in RA rats through Western blot and qPCR assays. The findings indicated that the expression levels of IL-33, sST2, and ST2L were markedly higher in RA group than those of control group (*P* < 0.01), whereas the levels were apparently lower in prednisone and GTW groups than model group (*P* < 0.01). In prednisone and GTW groups, protein expression levels of IL-33, sST2, and ST2L declined markedly in comparison to model group (*P* < 0.01). Furthermore, the protein expression levels of IL-33, sST2, and ST2L decreased greatly in the JWJGF groups administered at three doses compared with model group (*P* < 0.01) (Figure 3(a)). This indicated that JWJGF decreased the protein expression levels of IL-33, sST2, and ST2L in RA rats. Similarly, the same conclusion was obtained by qPCR (Figure 3(b)).

### 3.4. Effects of Liquiritigenin on RASF Cells

**Figure 4:**
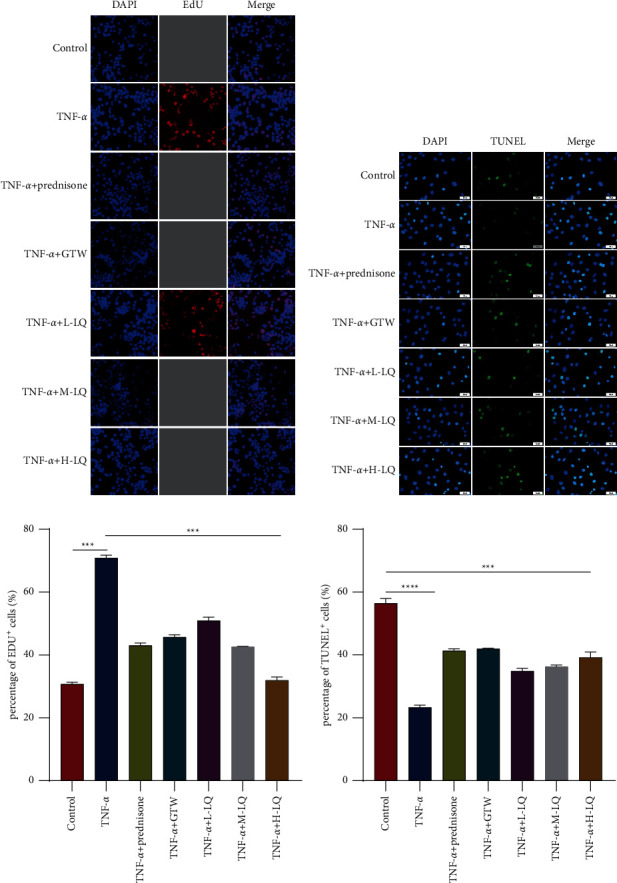
LQ action on RASF cell proliferation was detected using EDU assay and TUNEL assay. (a, b) Representative images and quantification. (c) Results of EdU assays. (d) Results of TUNEL assays. RASF cells were treated with TNF-*α* (10 ng/mL), LQ (50 *μ*M, 100 *μ*M, and 150 *μ*M), prednisone (200 ng/mL), and GTW (30 *μ*g/mL) for 24. (h) Data of the assays were expressed as mean ± SD (*n* ≥ 3). ^*∗*^*p* < 0.05, ^*∗∗*^*p* < 0.01, ^*∗∗∗*^*p* < 0.001, and ^*∗∗∗∗*^*p* < 0.001 based on either Student's *t*-tests for pairwise comparison or one-way ANOVA using Tukey's tests for multiple group comparison.

Using the network pharmacological approach, we found that LQ had the largest number of connections among all compounds. It is extracted from the stems of *Glycyrrhiza uralensis Fisch* and known as 7,4-dihydroxy-dihydroflavone with the chemical formula C_15_H_12_O_4_ and molecular weight of 256.25. As a dihydroflavonoid compound, LQ has certain effects on the cardiovascular system and nervous system. In addition, it has various pharmacological effects such as antiviral, antioxidant, antitumor, and estrogen effects. To figure out the effect of LQ on RASF cells, the effect of LQ on RASF cells was verified using EDU and TUNEL experiments. EDU results revealed that the positive EDU cell count was apparently elevated in TNF-*α* group compared with control group (*P* < 0.01), whereas that was decreased markedly in prednisone and GTW groups compared with TNF-*α* group (*P* < 0.01). Meanwhile, the positive EDU cell count in high-dose LQ group was substantially decreased compared with TNF-*α* group, indicating that LQ effectively inhibited the proliferation of RASF cells (Figure 4(a)). Simultaneously, TUNEL assay findings revealed that the positive TUNEL cell count in TNF-*α* group was markedly declined compared with control (*P* < 0.01), those in prednisone and GTW groups were substantially elevated in TNF-*α* group (*P* < 0.01), and those in high-dose LQ group were also greatly increased compared with TNF-*α* group, suggesting that LQ significantly promoted apoptosis of RASF cells. It was confirmed that LQ could inhibit the proliferation of RASF cells by EDU and TUNEL (Figure 4(b)).

### 3.5. Effect of Liquiritigenin on the IL33-ST2 Signaling Pathway in RASF Cells

**Figure 5:**
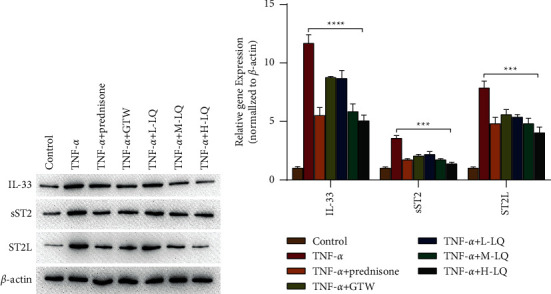
Detection of the effect of LQ on IL33-ST2 signaling pathway in RASF cells by Western blot and qPCR. (a) The relative protein expression levels of IL-33, sST2, and ST2L in all groups were subjected to Western blot. (b) The relative gene expressions of IL-33, sST2, and ST2L of each group were detected by qPCR. All data were presented as mean ± SD (*n* > 3 experiments). ^*∗*^*p* < 0.05, ^*∗∗*^*p* < 0.01, ^*∗∗∗*^*p* < 0.001, and ^*∗∗∗∗*^*p* < 0.0001 based on using Student's *t*-tests for pairwise comparison or one-way ANOVA using Tukey's tests for multiple groups.

To explore the effect of LQ on the IL33-ST2 signaling pathway of RASF cells, we verified its effect using Western blot and qPCR experiments. Western blot assay indicated a marked increase in expression levels of IL-33, sST2, and ST2L in TNF-*α* group compared with control group (*P* < 0.01), whereas a substantial decrease in expression levels of IL-33, sST2, and ST2L in prednisone group and GTW group was exhibited compared with TNF-*α* group (*P* < 0.01), and those in high-dose LQ group were also significantly decreased compared with TNF-*α* group (Figure 5(a)). These findings displayed that LQ effectively inhibited expressions of IL-33, sST2, and ST2L proteins, and the qPCR experiment confirmed the same results (Figure 5(b)).

## 4. Discussion

The main pathological manifestations of RA are synovial cell proliferation, synovial inflammation, and the formation of vascular opacities, which in turn erode cartilage and bone, ultimately leading to joint deformity and mobility impairment. The pathogenesis of RA is complex, involving genetic, environmental, and immune factors [12–14]. T cell-mediated autoimmunity is also a key factor in RA occurrence and CD4^+^ T cells have been reported to be associated with the pathogenesis of RA. At present, options for RA treatment have been improved from initial nonsteroidal anti-inflammatory drugs and traditional disease improvement antirheumatic drugs (cDMARDs) to targeted biologics and targeted small molecule drugs. Biological agents targeted for RA are mainly concentrated on various inflammatory factors downstream of pathogenesis including TNF-, IL-6, and IL-17 [15–17].

JWJGF is a long-term clinical study of the vaccine prescription of this research group presenting a satisfactory curative effect on RA treatment. To explore whether JWJGF was effective in RA treatment, we identified the key component (Liquiritigenin) and core target (IL-33) of JWJGF through network pharmacology. LQ is a compound extracted from the stems of *Glycyrrhiza uralensis Fisch*. As a dihydroflavonoid compound, LQ has certain effects on the cardiovascular system and nervous system. Besides, LQ has various pharmacological effects such as antiviral, antioxidant, antitumor, and estrogen effects. LQ indicated a role in inhibiting the proliferation of human cervical cancer HeLa cells and inducing cell apoptosis. LQ reduces vascular growth in transplanted tumors by inhibiting vascular endothelial growth factors and ultimately suppressing tumor progression. Kim had found that LQ can activate p53 and further activate p21, eventually causing the cervical cancer cell cycle to stagnate G1 and G2/M phases [18–21]. IL-33, derived from epithelial cells, is recognized as a cytokine of the IL-1 family. It can be combined with ST2 and regulate the secretion of inflammatory factors. IL-33 mainly originates from epithelial and endothelial cells and fibroblasts. It has dual biological roles like other IL-1 family members. It functions both as a transcription factor when localized in the nucleus and as a cytokine when secreted outside the cell. ST2 is expressed in many immune cells and nonconstructive blood cells as a high-affinity receptor of IL-33. IL-33 can also be released after stress and infection, which plays an important role in an allergic reaction, inflammation, autoimmune disease, and host defense. Lately, increasing studies have reported that IL-33 is closely related to heart-related diseases, diabetes, kidney disease, and autoimmune development. IL-33 binds to ST2 receptors to regulate inflammatory factor secretion [22–24].

To find out the molecular mechanism of JWJGF in RA treatment, we constructed a model of RA rats to verify the effects of JWJGF on synovial tissues, the infiltration of inflammatory cells, and chondrocyte injury. Compared with RA group, neither hyperemia, edema, nor synovial cell proliferation was reported in JWJGF group. Little infiltration of inflammatory cells was found.

Compared with mock group, expression levels of IL-1, IL-5, IL-6, IL-13, IL-17, IL-1*β* TNF-*α*, and TNF-*γ* in the high-dose JWJGF group were greatly declined compared with RA group (*P* < 0.01), indicating JWJGF could inhibit both synovial tissue proliferation and inflammation. To further explore the mechanism of JWJGF in the treatment of RA, we observed the effect of JWJGF on the IL33-ST2 signaling pathway in RA rats by Western blot and qPCR assays. The results presented a great decrease in protein expression levels of IL-33, sST2, and ST2L in JWJGF group (*P* < 0.01). This suggested that JWJGF might inhibit inflammation by inhibiting the IL33-ST2 signaling pathway.

Through network pharmacology, LQ was mostly connected with edges. To explore whether LQ acted on RASF cells, we observed the effect of LQ on RASF cells by EDU and TUNEL experiments. Findings of EDU assays indicated a sharp decrease in the number of positive EDU cells in the high-dose LQ group compared with TNF-*α* group. The results indicated that LQ significantly inhibited RASF cell proliferation. TUNEL assay findings indicated an apparent elevation in the number of positive TUNEL cells in the high-dose LQ group compared with TNF-*α* group. The results suggested that LQ significantly promoted RASF cell apoptosis. To further investigate LQ action on the IL33-ST2 signaling pathway in RASF cells, we compared effects of Liquiritigenin on the IL33-ST2 signaling pathway between high-dose LQ group and TNF-*α* group by Western blot and qPCR assays. The protein expression levels of sST2 and ST2L decreased substantially (*P* < 0.01). The results exhibited that LQ significantly inhibited the expressions of IL-33, sST2, and ST2L proteins, and the qPCR experiment confirmed the same findings. This suggested that Liquiritigenin might mediate the proliferation of RASF cells by inhibiting the IL33- ST2 signaling pathway.

JWJGF produces little toxicity, and it is safe to be applied clinically as per the prescribed dose and course of treatment. In the preliminary research of this research team, the toxicity of JWJGF was detected by performing acute and chronic toxicity tests. The acute toxicity test was designed as follows: 40 Kunming mice were randomly divided into a blank group and a JWJGF group, 20 in each group. The JWJGF group was administered at the maximum dose of 24.3 g/kg once, and the blank group was given an equal volume of distilled water. After 14 days, the mice were sacrificed and the main organs were harvested for pathological section comparison. The chronic toxicity test was designed as follows: a total of 80 rats were randomly divided into 4 groups, 20 in each group. A control group, JWJGF high-, medium-, and low-doses groups were prepared. The animals were given different concentrations of drugs by gavage for 6 d a week for 3 consecutive months. The control group was given an equal quantity of distilled water. The general manifestations of rats, poisoning reactions, and death before and after gavage were observed, and hemocytology and blood biochemical indexes, organ coefficients, and pathological examinations of main organs were recorded. The results in the acute toxicity experiment indicated that both groups of mice were generally stable following 2 to 14 d administration, with normal diets. No toxicity or death cases were reported. After anatomy, there were no abnormal changes in the organs. Pathological examinations revealed chronic inflammation in the partial trachea, and no obvious lesions were visualized in other organs. The chronic toxicity test revealed that the rats were administered with JWJGF at 10, 25, and 50 times of the recommended daily dose of adults in clinical practice by continuous intragastric administration for 3 months, and no death was reported. There were no obvious changes in general condition, weight, main organs, and hematological indexes of each drug group [25]. It is therefore obtained that the drug concentration selected in the present study on the basis of previous research is appropriate.

## 5. Conclusion

We firstly predicted possible targets and pathways of JWJGF for RA in a network pharmacology approach. The key component (LQ) and the core target (IL-33) of JWJGF were identified. Further, we found that JWJGF could inhibit the IL33-ST2 signaling pathway using Western blot and qPCR assays. Finally, JWJGF was verified as being responsible for inhibiting proliferation of RASF cells and the IL33- ST2 signaling pathway.

## Data Availability

The data used to support the findings of this study are included within the article.
